# Chemical and structural analysis of *Eucalyptus globulus* and *E. camaldulensis* leaf cuticles: a lipidized cell wall region

**DOI:** 10.3389/fpls.2014.00481

**Published:** 2014-09-16

**Authors:** Paula Guzmán, Victoria Fernández, José Graça, Vanessa Cabral, Nour Kayali, Mohamed Khayet, Luis Gil

**Affiliations:** ^1^Forest Genetics and Ecophysiology Research Group, School of Forest Engineering, Technical University of MadridMadrid, Spain; ^2^Instituto Superior de Agronomia, Centro de Estudos Florestais, Universidade de LisboaLisboa, Portugal; ^3^Mass Spectrometry Unit, Faculty of Chemistry, University Complutense of MadridMadrid, Spain; ^4^Department of Applied Physics I, Faculty of Physics, University Complutense of MadridMadrid, Spain

**Keywords:** cell wall, cuticle, cutin, eucalypt, leaf, lipids, polysaccharides, ultra-structure

## Abstract

The plant cuticle has traditionally been conceived as an independent hydrophobic layer that covers the external epidermal cell wall. Due to its complexity, the existing relationship between cuticle chemical composition and ultra-structure remains unclear to date. This study aimed to examine the link between chemical composition and structure of isolated, adaxial leaf cuticles of *Eucalyptus camaldulensis* and *E. globulus* by the gradual extraction and identification of lipid constituents (cutin and soluble lipids), coupled to spectroscopic and microscopic analyses. The soluble compounds and cutin monomers identified could not be assigned to a concrete internal cuticle ultra-structure. After cutin depolymerization, a cellulose network resembling the cell wall was observed, with different structural patterns in the regions ascribed to the cuticle proper and cuticular layer, respectively. Our results suggest that the current cuticle model should be revised, stressing the presence and major role of cell wall polysaccharides. It is concluded that the cuticle may be interpreted as a modified cell wall region which contains additional lipids. The major heterogeneity of the plant cuticle makes it difficult to establish a direct link between cuticle chemistry and structure with the existing methodologies.

## INTRODUCTION

The cuticle is an asymmetric, composite layer covering the surface of most aerial organs of plants (Tyree et al., 1990; Fernández and Brown, 2013), which is mainly extruded into the external wall of epidermal cells (Pollard et al., 2008; Javelle et al., 2010). Due to its location at the interface between the plant and the surrounding environment, the cuticle plays an array of crucial protective and physiological functions (Remus-Emsermann et al., 2011; Serrano et al., 2014). Additionally, it has a role in organ growth and differentiation (Kutschera and Niklas, 2007; Takada and Iida, 2014).

The multi-functional character of the plant cuticle is provided by its complex and heterogeneous chemical and physical nature (Gouret et al., 1993; Guzmán et al., 2014a). From a chemical viewpoint, the cuticle is generally defined as a cutin and/or cutan polymer matrix, with epicuticular waxes deposited on to its outer surface, embedded intracuticular waxes and phenolics, and polysaccharides stemming from the cell wall (Domínguez et al., 2011). Cutin is formed by a network of inter-esterified, hydroxy and hydroxy-epoxy C_16_ and/or C_18_ fatty acids (Kolattukudy, 1980), sometimes linked with glycerol (Graça et al., 2002). The chemical composition of an alternative cuticle matrix polymer named cutan remains controversial (Tegelaar et al., 1991; Deshmukh et al., 2005). Cuticular waxes (soluble cuticular lipids) are complex mixtures of aliphatic (mainly long-chain fatty acids, alcohols, alkanes, aldehydes, esters and ketones) and aromatic compounds (Jetter et al., 2006). Polysaccharides present in the cuticle are similar to those found in the cell wall (i.e., cellulose, hemicelluloses and pectins; López-Casado et al., 2007; Guzmán et al., 2014b). Based on the relative abundance of chemical constituents (Schmidt and Schönherr, 1982; Heredia, 2003; Samuels et al., 2008), the cuticle has been conceived as a hydrophobic membrane.

The cuticle has been generally described as having three different layers from the external to the internal surface, i.e.,: (i) the epicuticular wax (EW) layer, (ii) the cuticle proper (CP), and (iii) the cuticular layer (CL; von Mohl, 1847; Jeffree, 2006). It has been traditionally assumed that cutin/cutan and waxes are found both in the CP and CL, while the presence of polysaccharides is restricted to the CL (von Mohl, 1847; Jeffree, 2006). By contrast, the presence of cellulose and pectins in both cuticular layers has been recently reported (Guzmán et al., 2014b). For various species and organs, different patterns of cuticular ultra-structure have been categorized on the basis of transmission electron microscopy (TEM) observations (Holloway, 1982; Jeffree, 2006). However, a fair degree of cuticular structural variation may arise from e.g., the TEM sample preparation procedure implemented as recently shown by Guzmán et al. (2014a).

The cuticular chemical composition and/or ultra-structure of a few organs and species has been studied by gradual extraction and analysis of different cuticular fractions (e.g., Fernández et al., 2011, 2014; Tsubaki et al., 2013; Belge et al., 2014), and using diverse microscopic means (e.g., Canet et al., 1996; Casado and Heredia, 2001; Koch et al., 2004). The potential link between chemistry and structure of different cuticular fractions or specific molecular constituents has been examined chiefly focusing on EW (e.g., Baker, 1982; Barthlott et al., 1998; Jeffree, 2006). Integrative studies on the internal cuticle are however, scarce (e.g., Wattendorff and Holloway, 1982; Gouret et al., 1993; Viougeas et al., 1995; Yeats et al., 2012), and the existing relationship between chemical composition and structure remains unclear so far (Khayet and Fernández, 2012). In addition, there is currently a limited understanding of the potential cross-links and/or molecular assemblies taking place between cuticular chemical constituents (Pollard et al., 2008; Kunst and Samuels, 2009).

Given the chemical and physical complexity of the plant cuticle, there is a need for carrying out comprehensive investigations which may help us understand the cuticle in a holistic manner. The high complexity of the cuticle together with an array of technical constrains may lead to theoretical misconceptions concerning the structure, chemistry and function of the cuticle (Guzmán et al., 2014a,b). In this study we aimed at analyzing the link between ultra-structure and chemical composition (focusing on lipids) of the leaf cuticle, following an integrative approach. For this purpose *Eucalyptus camaldulensis* and *E. globulus* were used as model species due to their major importance for forestry production (Guzmán et al., 2013). Furthermore, despite such species are native to different habitats their taxonomic proximity may involve a certain degree of cuticular similarity. By merging different analytical and electron microscopy techniques before and after the selective extraction and identification of chemical constituents, the following questions were addressed: (i) is the adaxial leaf cuticle of both species structurally and chemically similar?, (ii) how does the gradual extraction of cuticular components affect cuticular ultra-structure as compared to intact tissues?, and (iii) is it currently possible to establish a relationship between cuticular chemical composition and structure?

## MATERIALS AND METHODS

### PLANT MATERIAL AND CUTICLE ISOLATION

The adaxial surface of fully expanded, mature, undamaged leaves of *E. camaldulensis* Dehn and *E. globulus* Labill was analyzed either intact or after enzymatic isolation of the cuticle. Leaves were collected at late summer from the apex of external, west-facing shoots of 30–35 years old trees growing at the Forest Engineering School Arboretum (Technical University of Madrid, Spain). For each species, 6 and 30 leaves were selected for intact leaf and isolated cuticle analyses, respectively.

Prior to cutting the central part of the leaf blades in small pieces for cuticle isolation, leaf margins and midribs were removed with a scalpel. Leaf cuticles were subsequently isolated in a solution containing 5% cellulase and 5% pectinase (Novozymes, Bagsvared, Denmark), plus 1% polyvinylpyrrolidone (Sigma–Aldrich, Munich, Germany) and 2 mM sodium azide, which was set to pH 5.0 by adding sodium citrate (Guzmán et al., 2014a). Tissues were maintained in solution (changed after 2 weeks) at room temperature (23–25^∘^C) for 1 month, manually shaking the flasks at frequent time intervals. After the extraction period, clean intact adaxial cuticles were selected, thoroughly washed in deionized water, air-dried, and stored for further use.

### EXTRACTION AND DETERMINATION OF SOLUBLE COMPOUNDS

Isolated cuticles were cut into small pieces, wrapped in filter paper and successively extracted in 150 mL of dichloromethane (6 h; Sigma–Aldrich), ethanol (12 h; Sigma–Aldrich) and distilled water (24 h) using a Soxhlet apparatus. Solvents were subsequently evaporated to dryness with a rotary evaporator and the material extracted was weighed and stored. Soxhlet extracted (SE) cuticles were also stored for further analysis.

The cuticular material extracted in dichloromethane was analyzed by gas chromatography-mass spectrometry (GC-MS). Samples were filtered using polytetrafluoroethylene (PTFE) filters prior to injection in a GC-MS apparatus (GC Varian CP-3800 coupled to a MS ion trap Varian Saturn 2200, GC-ITMS, automatic injector COMBI-PAL; CTC Analytics, Zwingen, Switzerland). The chromatographic conditions were as follows (86 min per run): injection volume 1 μL, with Helium as carrier gas (1.2 mL min^-1^), and injector temperature 290^∘^C. The column (SLB-5 ms fused silica capillary column, 30 m × 0.25 mm × 0.25 μm film thickness; Supelco, Sigma–Aldrich) was set to 40^∘^C (3 min), heating rate of 10^∘^C min^-1^ up to 290^∘^C (18 min), and 20^∘^C min^-1^ up to 310^∘^C (13 min). The MS conditions were: 70 eV ionization voltage, 150^∘^C trap temperature, 20–550 units of mass scan range with 8 min solvent delay. The compounds were identified and quantified by comparing their mass spectra with NIST library spectra and analyzing the corresponding peaks of GC-MS ion chromatograms.

### EXTRACTION AND DETERMINATION OF CUTIN CONSTITUENTS

For the two species, 80 mg of dehydrated (oven-dried at 40^∘^C before use), SE cuticles were used for cutin depolymerization. Samples were refluxed in 5 mL of 0.1 M sodium methoxide solution (Sigma–Aldrich) in methanol by heating in an oil bath at 80^∘^C for 3 h, and continuous stirring. For separation of de-esterified (filtered) and residual (non-filtered) material from the methanolysis process, mixtures were vacuum filtered using 1 μm pore PTFE filters. The residual material was oven-dried under vacuum at 40^∘^C overnight, weighted for the determination by mass-loss, of the total content of cuticle de-esterified material (one replicate) and saved for further examination.

The total volume of de-esterified material was divided for glycerol and cutin monomer and precursor identification (1:4, v:v), and handled separately. For glycerol analysis, the de-esterified aliquot was sampled as such. For cutin monomer identification, the respective aliquot was acidified to pH 6 with 0.05 M sulfuric acid in methanol, and the solvent was subsequently removed in a rotary evaporator at 50^∘^C. The obtained residue was partitioned in dichlorometane:water (1:1, v:v; 35 mL each) using a separating funnel. The upper aqueous, clear phase was discarded and the organic, opaque phase located below was partitioned again with the same volume of water.

For GC-MS determination, aliquots (1 mL) of the de-esterified material and of the accumulated organic phases were collected. Aliquots were dried under a N_2_ flow and derivatized with *N,O*-bis(trimethylsilyl)trifluoroacetamide with 1% trimethylchlorosilane (BSTFA with 1% TMCS, Sigma–Aldrich) and pyridine at a 1:1 (v:v) ratio. After 15 min staging in an oven at 60^∘^C, solutions were analyzed with an Agilent 5973 MSD GC-MS (Agilent, Santa Clara, CA, USA) in a DB-5 ms column (60 m, 0.25 mm i.e., 0.25 μm film thickness; Agilent), with the following GC conditions: injector 320^∘^C, initial oven temperature 100^∘^C (5 min), heating rate of 8^∘^C min^-1^ up to 250^∘^C, and 2.5^∘^C min^-1^ up to 320^∘^C (20 min). The MS conditions were: source 220^∘^C, quadrupole 150^∘^C, electron ionization energy 70 eV. Cutin monomers were identified by library search (NIST 98, Wiley 6), comparison to standards and/or mass spectra interpretation (Graça et al., 2002). Monomer quantification was calculated from the areas of the corresponding peaks in the CG-MS ion chromatograms. As internal standards for glycerol and cutin monomers, we used 1,12-dodecanediol (0.05–0.3 mg; ≥98.5% GC, Sigma–Aldrich) and methyl 12-hydroxystearate (0.05–0.1 mg; ≥99.0% GC, Sigma–Aldrich), respectively. A response factor of 0.33 in relation to the internal standard (1,12-dodecanediol) was used for the quantitation of glycerol. The internal standards were diluted in methanol, homogenized in a warm bath, and added to the de-esterified material before derivatization (Graça and Lamosa, 2010).

### ELECTRON MICROSCOPY

Intact leaves, isolated cuticles, SE cuticles, and the residues obtained after de-esterification of SE cuticles (subsequently called SED residues) were examined by TEM and scanning electron microscopy (SEM).

Gold-sputtered adaxial leaf surfaces and both the inner and outer cuticle and residue surfaces (three replicates per sample) were observed with a Philips XL30 SEM (Eindhoven, The Netherlands) microscope.

For TEM observation, samples were cut into 4 mm^2^ pieces and fixed in 2.5% glutaraldehyde-4% formaldehyde freshly prepared from paraformaldehyde (both from electron microscopy sciences (EMS), Hatfield, PA, USA) for 6 h at 4^∘^C, rinsed in ice-cold phosphate buffer, pH 7.2, four times within a period of 6 h and left overnight. Tissues were post-fixed in a 1:1 2% aqueous osmium tetroxide (TAAB Laboratories, Berkshire, UK) and 3% potassium ferrocyanide (Sigma–Aldrich) solution for 1.5 h. They were then washed with distilled water (x3), dehydrated in a graded series of 30, 50, 70, 80, 90, 95, and 100% acetone (x2, 15 min each concentration) and embedded in acetone-Spurr’s resin (TAAB Laboratories) solutions [3:1, 2 h; 1:1, 2 h; 1:3; 3 h (v:v)] and in pure resin overnight at room temperature (25^∘^C). Tissues were finally embedded in blocks which were incubated for 3 days at 70^∘^C. Ultra-thin sections of three leaf and cuticle pieces per treatment were cut, mounted on nickel grids and post-stained with Reynolds lead citrate (EMS) for 5 min. They were subsequently observed with a Jeol 1010 TEM (Tokyo, Japan) operated at 80 kV and equipped with a CCD megaview camera.

The thickness of entire cuticle transversal sections and of the different cuticular regions was measured on TEM micrographs of leaves, intact and SE cuticles, and SED residues using siViewer (Olympus Soft Imaging Solutions, Münster, Germany) and ImageJ 1.45s (National Institutes of Health, Bethesda, MD, USA) softwares. Measurements were performed at periclinal areas, excluding zones located in the vicinity of cuticular pegs, and also the EW layer. Micrographs corresponding to extremely thick or thin cuticle sections or with no clear limits between adjacent cuticular regions were discarded. Finally, 15 micrographs of each sample (corresponding to three 4 mm^2^ cuticle pieces) were selected for thickness measurement, with 10–20 repetitions.

### FOURIER TRANSFORM INFRARED (FTIR) SPECTROSCOPY

Infrared spectra of cuticle samples (i.e., intact, SE cuticles, and SED residues), were obtained with an attenuated total reflectance (ATR) accessory (MIRacle ATR; PIKE Technologies, Madison, WI, USA) coupled to a FTIR spectrometer (Nicolet Nexus 670–870; Thermo Fisher Scientific, Waltham, MA, USA). Spectra of intact samples were recorded in transmission mode in the range 4000–400 cm^-1^ with 4 cm^-1^ resolution and accumulating 64 scans, and analyzed with Omnic v4.1b (Thermo Fisher Scientific) software. Two replicates per sample were analyzed.

## RESULTS

### ELECTRON MICROSCOPY OBSERVATIONS

#### Intact leaves and cuticles

**FIGURE 1:**
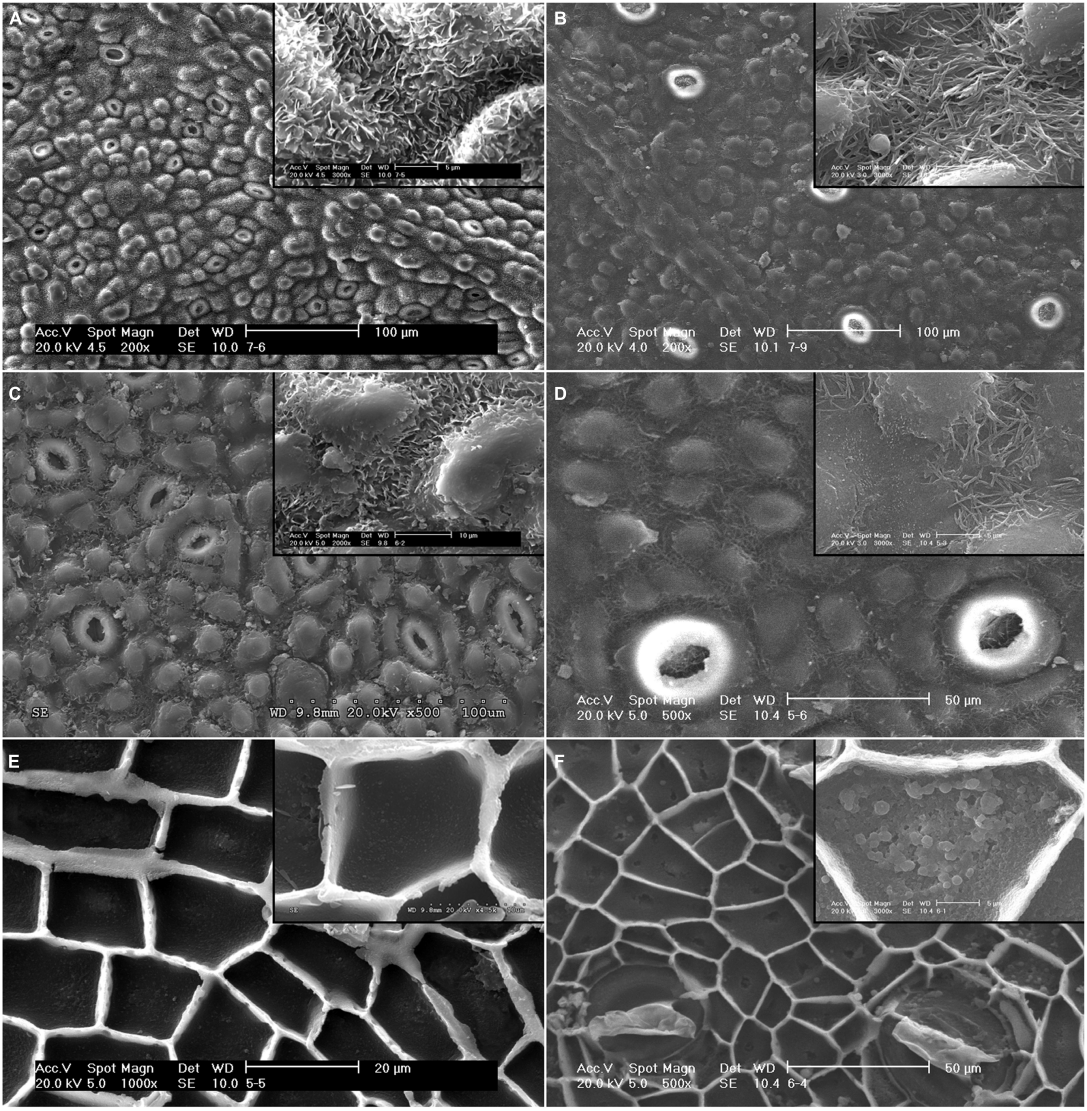
**Scanning electron microscopy (SEM) micrographs of the adaxial leaf surface and intact, isolated cuticles of *E. camaldulensis* and *E. globulus*.** Surface and detail of EW structures of *E. camaldulensis* **(A)** and *E. globulus* leaves **(B)**. Outer side and detail of EW structures of *E. camaldulensis* **(C)** and *E. globulus* cuticles **(D)**. Inner side and detail of the granules in the periclinal cuticle of *E. camaldulensis* **(E)** and *E. globulus* cuticles **(F)**. Bars in insert images: 5 μm **(A,B,D,F)**, 10 μm **(C,E)**.

The adaxial surface of *E. camaldulensis* and *E. globulus* leaves and intact cuticles had a rough topography conferred by EW and epidermal structures such as papillae and stomata (**Figures 1A–D**). The most prominent EW structures were plates with different orientations for *E. camaldulensis* and tubes for *E. globulus*. Plates were observed to densely cover the whole leaf and cuticle surfaces with the exception of the tip of some papillae (**Figures 1A,C**), while wax tubes were principally found around papillae and stomata (**Figures 1B,D**). The isolated cuticle topography was smoother compared to that of intact leaves, chiefly due to the loss or distortion of some EW during the cuticle isolation process (**Figures 1C,D**). The cuticle inner sides revealed the epidermal cell surface shape (i.e., the upper periclinal wall with papillae, and the anticlinal flanges or cuticular pegs; **Figures 1E,F**), and showed a granular pattern especially noticeable in *E. globulus* (**Figure 1F**).

**FIGURE 2:**
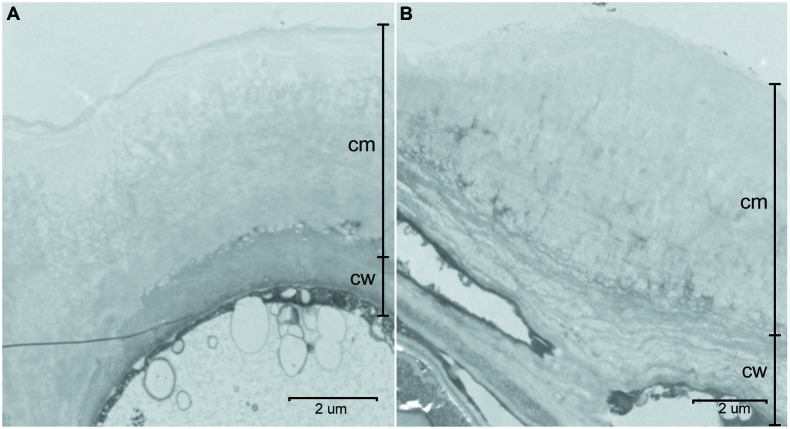
**Transversal sections of the adaxial cuticle and outer cell wall of *E. camaldulensis***(A)** and *E. globulus***(B)** intact leaves.** cm: cuticular membrane, cw: cell wall. Bars: 2 μm.

**FIGURE 3:**
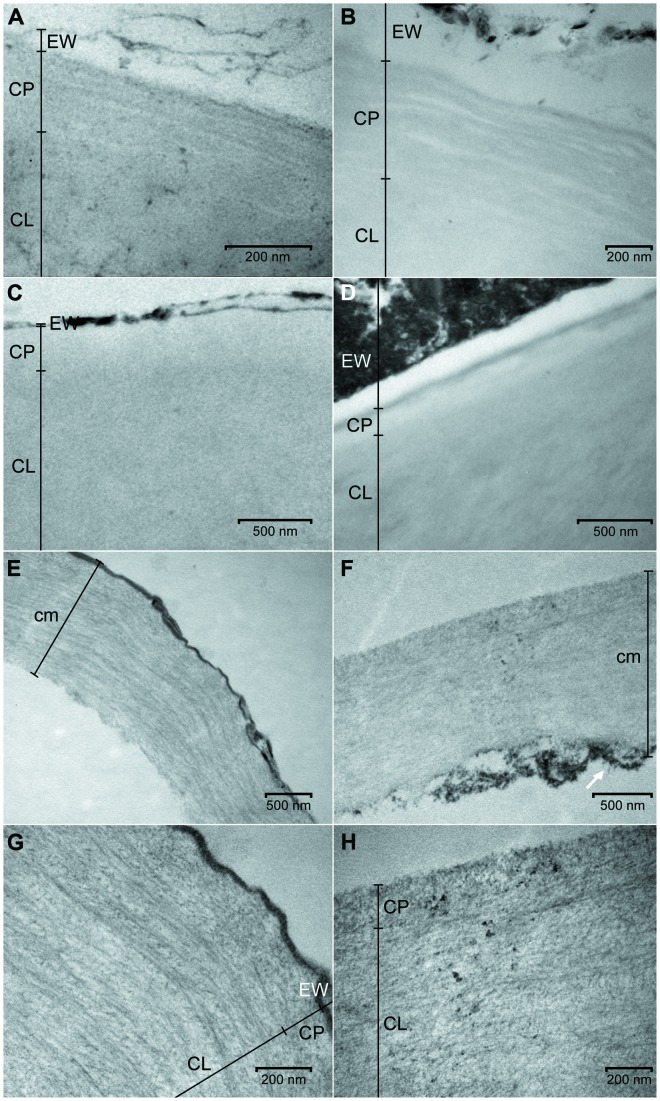
**Transmission electron microscopy (TEM) micrographs of the adaxial leaf cuticles of *E. camaldulensis* and *E. globulus* both intact and after successive extractions.** Outer regions of *E. camaldulensis* **(A)** and *E. globulus* **(B)** intact cuticles. Outer regions of *E. camaldulensis* **(C)** and *E. globulus* **(D)** SE cuticles. Complete transversal section and detail of the outer region of *E. camaldulensis* **(E,G)** and *E. globulus* **(F,H)** SED residue. SE: Soxhlet extracted, SED: Soxhlet extracted, and depolymerized. CL: cuticular layer; CP: cuticle proper; cm: cuticular membrane; EW: epicuticular wax layer. Bars: 200 nm **(A,B,G,H)**, 500 nm **(C–F)**. The arrow indicates an area of ripped cellulose fibrils as an example **(F)**.

The cuticles of *E. globulus* and *E. camaldulensis* were between 5.5 to 9.5 μm and 2.5 to 5.0 μm thick, respectively (**Figure 2**). The CP of *E. camaldulensis* (100–250 nm thick; **Figure 3A**) and *E. globulus* (350–500 nm thick; **Figure 3B**) cuticles represented between 5 and 6% of the total cuticle thickness. The cuticle of *E. globulus* was about two times thicker and had a two to three times thicker CP than *E. camaldulensis*. The thickness of the EW layer was not considered since the TEM sample preparation procedure may significantly affect EW composition and structure.

In contrast to the EW layer, the remaining cuticular ultra-structure was not significantly altered by the isolation process as derived from comparing intact cuticle TEM micrographs either as isolated tissues or when attached to the leaf. According to the nomenclature suggested by Holloway (1982), the cuticle of both species had a faintly lamellate CP, with alternate electron-lucent and electron-dense lamellae (**Figures 3A,B**). While the CL of *E. camaldulensis* cuticle showed a gradual decrease of the reticulum toward the CP, an amorphous region could be identified between the CP and the reticulate inner region in the cuticle of *E. globulus*. The reticulate and amorphous CL areas close to the CP can be respectively, observed in **Figures 3A,B**.

#### Soxhlet extracted cuticles

**FIGURE 4:**
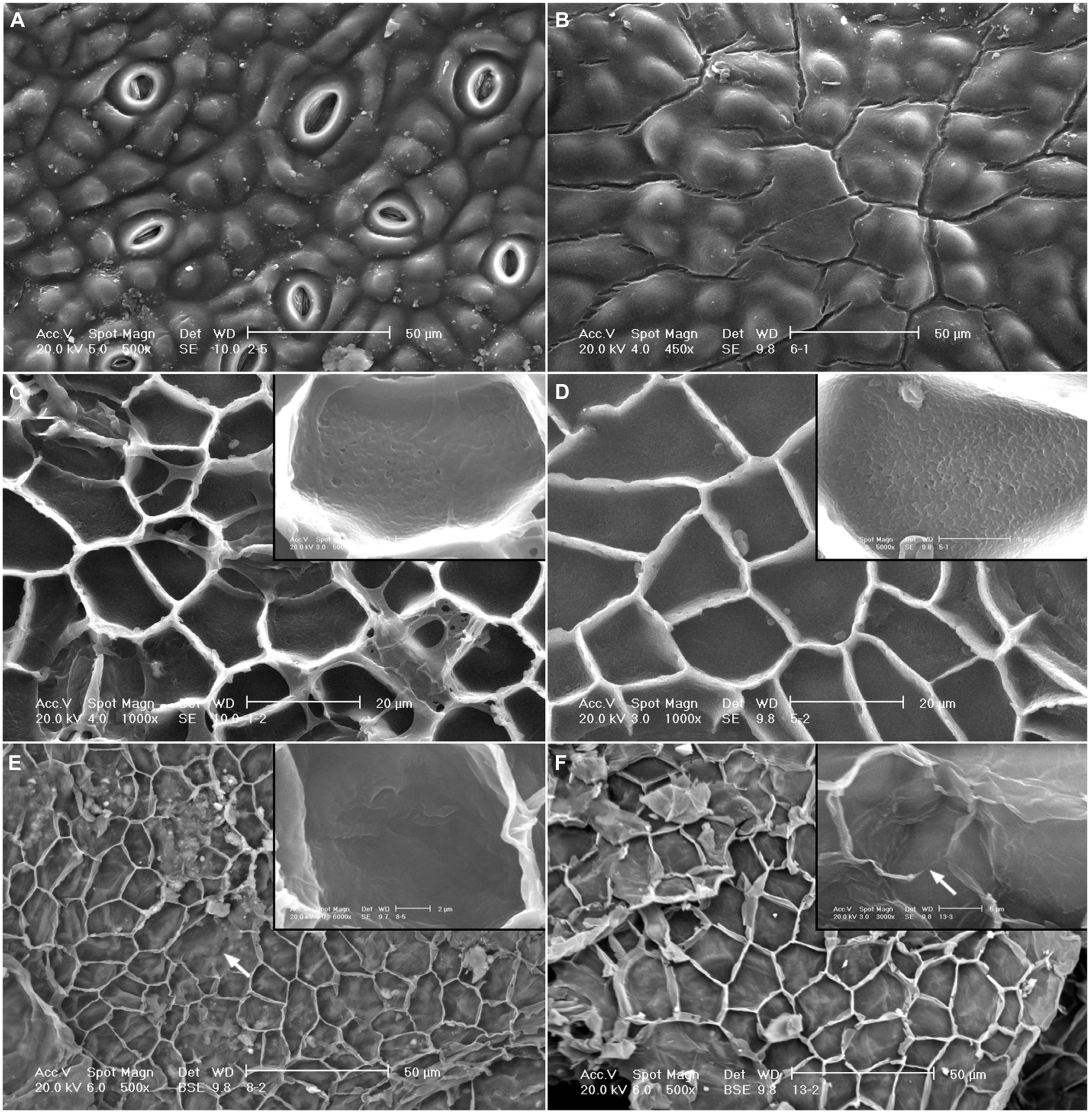
**SEM micrographs of the adaxial leaf cuticles of *E. camaldulensis* and *E. globulus* after successive extractions.** Outer side of *E. camaldulensis* **(A)** and *E. globulus* **(B)** SE cuticles. Inner side and detail of *E. camaldulensis* **(C)** and *E. globulus* **(D)** SE cuticles. Inner side and detail of *E. camaldulensis* **(E)** and *E. globulus* **(F)** SED residues. SE: Soxhlet extracted, SED: Soxhlet extracted, and depolymerized. Bars in insert images: 5 μm **(C,D,F)**, 2 μm **(E)**. Arrows indicate areas were the anticlinal cuticle was not visible **(E,F)**.

The topography of the outer surface of SE cuticles became smoother compared to intact tissues (**Figures 4A,B**). While most of the EW were solubilized, some plates remained in *E. camaldulensis* SE cuticles (**Figure 4A**), and a cracked EW layer was observed in *E. globulus* SE cuticles (**Figure 4B**). Whilst no major structural changes were noticeable in the inner side of SE cuticles, the granules observed in intact cuticles were mostly removed, and cavities with a similar size were identified in SE periclinal cuticle areas (**Figures 4C,D**).

Patches of EW crystalline projections were also observed in cuticular cross-sections. For both species, the CP lamellae disappeared after Soxhlet extraction. After this process an amorphous, electron-lucent band arose which had a thickness slightly below or within the range of the intact CP. The upper limit of this band was a relatively thin, discontinuous, electron-dense line, which may correspond to non-solubilized waxes (**Figures 3C,D**). The Soxhlet extraction process did not seem to modify the CL reticulum, and no remarkable structural or thickness changes in the rest of this region were observed (data not shown). The presence of granules in the inner surface of the cuticle was also noticed in TEM micrographs (data not shown).

#### De-esterified residues

The structure of SED residues was disrupted as observed in the inner side of such tissues by SEM (**Figures 4E,F**). The concavity of the periclinal cuticle appeared to increase (e.g., **Figure 4E**), while the anticlinal cuticle seemed to be more fragile and was not distinguishable in some areas (**Figures 4E,F**). The upper side of SED residues had a similar appearance to that of SE cuticles (data not shown).

A considerable cuticle thickness decrease of approximately 60–75% and 70–80% for *E. camaldulensis* and *E. globulus* cuticles was observed as a result of the de-esterification process (**Figures 2** vs. **3E,F**). This reduction largely occurred in association with the inner cuticle part (i.e., the zones which were closer to the epidermal cell walls). This was evidenced by the longer cuticular pegs observed in SED residues as compared to those of intact and SE cuticles (data not shown) and by the presence of EW (e.g., **Figures 3E,G**).

The cell wall architecture was observed in the transversal sections of SED residues (**Figures 3E–H**). While diffuse cellulose fibrils were detected in the outermost cuticle region, a lamellate, helicoidal cellulose pattern was noticed in the adjacent inner region. The position within the cuticle of these regions corresponded to that of the CP and CL (**Figures 3G,H**). The outer, diffuse regions had a thickness similar or sometimes slightly below that of the CP of intact cuticles, as also recorded for the amorphous outer region of SE cuticles. The cellulose lamellae of the inner region had a mainly parallel orientation to both the periclinal (**Figures 3E–H**) and the anticlinal cell wall underneath (data not shown), which was especially noticeable in *E. camaldulensis* SED residues (**Figures 3E,G**). The electron-dense line located in the EW layer position seemed not to be affected by the de-esterification process, especially in *E. camaldulensis* (**Figures 3E,G**). The cellulose fibrils localized in the innermost cuticle areas of SED residues appeared ripped to some extent (e.g., **Figure 3F**).

#### Cuticle chemical composition

**FIGURE 5:**
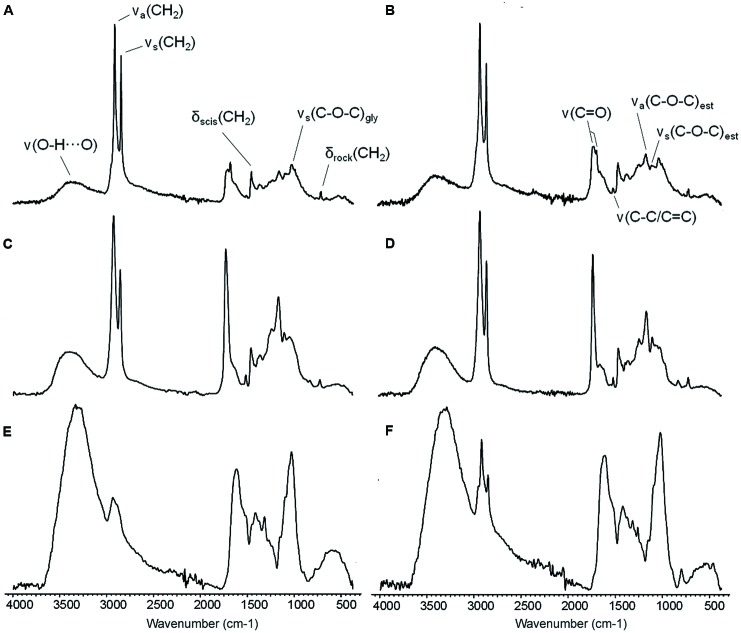
**ATR-FTIR spectra of the adaxial leaf cuticles of *E. camaldulensis***(A,C,E)** and *E. globulus***(B,D,F)**.** Intact cuticles **(A,B)**, SE cuticles **(C,D)**, and SED residues **(E,F)**. SE: Soxhlet extracted, SED: Soxhlet extracted and depolymerized. ν: stretching, δ: bending, a: asymmetric, s: symmetric, scis: scissoring, rock: rocking, est: ester, gly: glycosidic bond.

The relative proportion of cuticular chemical constituents obtained after successive extractions are shown in **Table 1**. Both eucalypt species followed a similar trend concerning the relative amount of chemical constituents. The de-esterified material represented the greatest percentage, accounting for approximately half of the total cuticle weight, followed by Soxhlet extracted, soluble compounds. The remaining SED residues (mainly polysaccharides; **Figures 5E,F**) constituted the smallest cuticular fraction.

**Table 1:** Chemical composition of adaxial cuticles of *Eucalyptus camaldulensis* and *E. globulus* leaves after successive extractions expressed as weight percentages of the intact cuticles.

Sample	Soxhlet extractives (%)	SE cuticle	
		De-esterified material (%)	Residual material (SED residue; %)
*E. camaldulensis*	38.3	48.2	13.5
*E. globulus*	37.3	53.6	9.1

*SE, Soxhlet extracted; SED, Soxhlet extracted and depolymerized.*

The dichloromethane-soluble extractives included aromatic and chiefly aliphatic compounds. Aliphatic molecules accounted for approximately 96% and 76% of the total amount of identified compounds for *E. camaldulensis* and *E. globulus* cuticles, respectively. The relative amount of aromatics was hence six times greater in *E. globulus* than in *E. camaldulensis*. Cyclic compounds (both alicyclics and aromatics) constituted about 39% (*E. camaldulensis)* and 76% (*E. globulus)*. Among *E. camaldulensis* extractives, the most abundant soluble compound types were aliphatic esters (26%), followed by primary alcohols (20%). For *E. globulus*, terpenes and terpenoids represented the highest percentage of extractives (45%, chiefly sesqui- and tri-terpenoids), followed by phenolics (21%). Individually, [(Z)-octadec-9-enyl] (Z)-octadec-8-enoate (22%) and hentetracontan-1-ol (16%) were the most abundant soluble lipids identified in *E. camaldulensis* cuticle. (1aR,4R,4aR,7R,7aS,7bS)-1,1,4,7-tetramethyl-2,3,4a,5,6,7,7a,7b-octahydro-1aH-cyclopropa[e]azulen-4-ol (16%) and 5-methoxy-2,2-dimethyl-10-(3-methylbut-2-enyl)pyrano[3,2-g]chromen-8-one (13%) constituted the highest percentages of soluble extractives for *E. globulus* cuticle.

For the two species, the lipidic, de-esterified material (**Table 2**) was found to be chiefly composed of aliphatic ω-hydroxyacids, which represented approximately 62% (*E. camaldulensis*) and 69% (*E. globulus*) of the total amount of such material (also including unidentified long-chain aliphatic compounds). In both samples α,ω-diacids were also identified and amounted to 17% (*E. camaldulensis*) and 8% (*E. globulus*). For the two species, the number and total amount of C_18_ cutin monomers were higher than those of C_16_ monomers, with C_18_/C_16_ ratios of 1.6 (*E. camaldulensis*) and 3.1 (*E. globulus*). Hydroxylated monomers accounted for 76 and 72% of the total amount of compounds for *E. camaldulensis* and *E. globulus* samples. Among them, those having two hydroxyl groups were the most abundant (42%) in *E. camaldulensis*, followed by the identified three-hydroxyl-compound (22%, **Table 2**). By contrast, monomers having one hydroxyl group accounted for the greatest percentage (30%) in *E. globulus*, closely followed by those having two hydroxyl groups (28%). On the other hand, epoxy monomers constituted about 13 and 35% for *E. camaldulensis* and *E. globulus* de-esterified material, respectively. The amount of hexadecanoic acid was relatively low as well as that of coumaric acid, the latter being only identified in *E. camaldulensis*. Although representing a minor contribution, glycerol was present in both samples and its amount was twofold for *E. camaldulensis*.

**Table 2:** Cutin composition of isolated adaxial cuticles of*E. camaldulensis* and *E. globulus* leaves.

	Relative abundance (%)	
Cutin components	*E. camaldulensis*	*E. globulus*
16-Hydrohexadecanoic acid	2.4	1.3
9(7–10),16-Dihydroxyhexadecanoic acid	28.1	17.6
9(10),18-Dihydroxyoctadecenoic acid^1^	0.0	5.1
9-Epoxy-18-hydroxyoctadecanoic acid	9.5	28.6
9-Epoxy-octadecane-1,18-dioic acid	3.0	4.8
9,10-Dihydroxyoctadecane-1,18-dioic acid	14.0	3.3
Epoxy-9(10),18-dihydroxyoctadecanoic acid^2^	0.0	1.7
9,10,18-Trihydroxyoctadecanoic acid	22.0	14.4
Coumaric acid	0.6	0.0
Hexadecanoic acid	0.4	0.2
Glycerol	4.4	2.4
Unidentified^3^	15.6	20.6

*^1^Position of double bond not assigned*

*^2^Position of epoxide not assigned*

*^3^Several long-chain aliphatic compounds*

#### FTIR spectroscopy

The ATR-FTIR spectra of intact and SE cuticles, and of SED residues are shown in **Figure 5**. The spectra of *E. camaldulensis* and *E. globulus* intact cuticles (**Figures 5A,B**) indicate the presence of long-chain linear aliphatic compounds which were associated with the intense and thin bands at 2916 and 2917 cm^-1^ and at 2849 cm^-1^ [asymmetric and symmetric vibrations of the methylene groups, CH_2_; ν_a_(CH_2_) and ν_s_(CH_2_), respectively], as well as smaller bands at 1462 and 1463 cm^-1^ and at 719 cm^-1^ [CH_2_ scissoring and rocking vibrations; δ_scis_(CH_2_) and δ_rock_(CH_2_), respectively]. By comparing the intensities of these bands with those corresponding to the ester groups in the spectra of intact and chiefly SE cuticles (**Figures 5C,D**), it could be reckoned that these aliphatic compounds are chiefly ascribed to soluble lipids.

The absorptions at 1713 and 1719 cm^-1^ and their shoulders at 1686 and 1687 cm^-1^ [C = O stretching vibration of the carbonyl bonds; ν(C = O)] for *E. camaldulensis* and *E. globulus* may correspond to soluble compounds with ketone or aldehyde functional groups which were identified in both samples. These vibrations together with the vibrations at 1168 cm^-1^ and at 1105 and 1104 cm^-1^ [asymmetric and symmetric C–O–C stretching vibrations of the ester bonds; ν_a_(C–O–C)_est_ and ν_s_(C–O–C)_est_, respectively] may also be associated with esterified soluble lipid compounds and especially with the cutin in the polymer form.

The low intensity bands at 1517 and 1518 cm^-1^ were related to the stretching of aromatic rings [ν(C–C) conjugated with (C = C) or ν(C-C)/(C = C)]. The middle intensity bands at 1030 and 1035 cm^-1^ were assigned to glycosidic bonds typical of polysaccharides [ν(C–O–C)_gly_]. The broad bands appearing at 3388 and 3345 cm^-1^ [H-bonded, O–H stretching vibration; ν(O-H…O)] were assigned to hydroxyl functional groups and could be related to tissue hydration. The assignment of other minor bands can be found in Heredia-Guerrero et al. (2014).

The spectra of SE cuticles (**Figures 5C,D**) showed an increment in the bands associated with ester functional groups. By comparing the intensity of the bands corresponding to the asymmetric and symmetric vibrations of CH_2_ and that of the ester C–O stretching vibration, it can be concluded that most of the CH_2_ can be assigned to cutin. The higher intensity of the bands associated with aromatic compounds in SE cuticles spectra as compared to those observed in the spectra of intact cuticles indicated the presence of aromatic compounds related to insoluble lipids and phenolics.

Finally, the spectra of SED residues (**Figures 5E,F**) exhibited an increase of the bands assigned to polysaccharides. In addition, the broad bands corresponding to hydroxyl groups increased with respect to the intact and SE cuticle spectra, and may correspond to hydrated polysaccharides. On the other hand, the spectra of *E. globulus* SED residue still showed the presence of a relatively minor amount of non-solubilized long-chain aliphatic compounds (**Figure 5F**), which constitute the most noticeable difference between the two species as derived from all the spectra analyzed.

## DISCUSSION

In this study, we analyzed the chemical composition of the adaxial leaf cuticle of *E. camaldulensis* and *E. globulus* in relation to their ultra-structure, by merging different analytical and microscopic methods. Such species were selected as model due to their commercial significance for forestry production. Since they belong to the same genus, such leaf cuticles may additionally have chemical and structural similarities which may however, be affected by their different native habitat. Only few studies analyzed plant cuticles following an integrative approach, and the link between cuticle chemistry and structure remains unclear. On the other hand, the presence and role of cuticular polysaccharides has been taken into account only in few investigations (e.g., Matas et al., 2004; López-Casado et al., 2007; España et al., 2014; Guzmán et al., 2014b).

## CUTICLE CHEMICAL COMPOSITION

According to the assignment of cuticular fractions after successive chemical extractions, cutin (directly associated with de-esterified material; e.g., España et al., 2014) was the most abundant component for the two eucalypt samples analyzed, accounting for 48–54% of the total cuticle weight, followed by soluble lipids (organic solvent extractives; about 38–37%). Polysaccharides actually represented the lowest cuticle fraction (the residual material; 14.9%).

Nevertheless, the different cuticle extraction processes may lead to significant chemical losses and to a potentially inaccurate assignment of chemical groups. For example, it is likely that the polysaccharide fraction is currently underestimated, in contrast to the possibly overestimated cutin fraction. The de-esterification process may have released additional chemical constituents which are linked by ester bonds (e.g., waxes, phenolics, pectins, or hemicelluloses), trapped in or strongly bound to cutin and/or polysaccharide networks. The constituents located in the inner cuticle regions were especially affected, as concluded from the estimated thickness loss and occurrence of ripped cellulose fibrils in SED residues. Furthermore, significant amounts of pectins, hemicelluloses, mineral elements and even cellulose may be lost when keeping tissues in aqueous solution, e.g., during the cuticle isolation, Soxhlet extraction, or de-esterification processes. A number of factors associated with e.g., the cuticle isolation process, solubility of different lipid types in organic solvents, and/or physical constraints that may limit the access of the solvents and solutes employed for lipid extraction and cutin depolymerization, can ultimately influence the gross amount, quality and structure of cuticular components.

Some of the Soxhlet extracted compounds identified in this study have been previously reported for *E. camaldulensis* and *E. globulus* (e.g., Li et al., 1997; Silvestre et al., 1997; Jeffree, 2006; Steinbauer et al., 2009; Verdeguer et al., 2009). Differences in relation to the occurrence and relative content of molecular constituents and soluble compound types were observed between the two species. Aliphatic esters (26%) and terpenes and terpenoids (45%) were the most abundant compounds in *E. camaldulensis* and *E. globulus* cuticles, respectively. The amount of cyclic compounds was almost twofold higher for *E. globulus* as compared to *E. camaldulensis* (76 vs. 39%), with aromatics being six times more abundant in *E. globulus* (24 vs. 4%).

The dissimilar cutin monomer composition of the two eucalypt species analyzed may lead to different cutin molecular architectures (Graça and Lamosa, 2010). This was however, not noticeable in terms of different TEM cuticular ultra-structures. *E. camaldulensis* had a higher proportion of C_18_ chain-length monomers as compared to *E. globulus* (C_18_/C_16_ ratio of 3.1 vs. 1.6, respectively). Additionally, *E. camaldulensis* had higher occurrence of monomers with mid-chain hydroxyl groups and a higher relative content of α,ω-diacids (17.0% vs. 8.1%) and glycerol (4.4% vs. 2.4%). The cuticle composition differences recorded may lead to structural differences concerning e.g., the degree of reticulation or molecular arrangement, also considering the occurrence of higher glycerol and α,ω-diacids amounts in *E. camaldulensis*, as observed in suberins (Graça et al., 2002; Graça and Lamosa, 2010).

## INTACT CUTICLE ULTRA-STRUCTURE

The ultra-structure of *E. camaldulensis* and *E. globulus* cuticles is characterized by having an EW layer with different crystalline projections (plates and tubes, respectively), a faintly lamellate CP and a CL with variable morphology. A fully reticulate CL was observed in *E. camaldulensis* cuticle, whereas an external amorphous CL was found between the CP and the reticulate internal CL of *E. globulus*. Intact cuticle inner surfaces were additionally covered with granular structures. The occurrence of a bi-layered CL, as observed for *E. globulus*, has been shown in other young and mature leaf cuticles of different species (e.g., Schmidt and Schönherr, 1982; Wattendorff and Holloway, 1982; Jeffree, 2006). The presence of granules in the inner surface of isolated leaf cuticles has been previously reported (Oladele, 1983; Carpenter et al., 2007).

In this study, we found inter-specific differences in relation to the thickness, ultra-structure, and chiefly chemical composition of the two eucalypt cuticles analyzed. Such cuticle variations may be associated with genetic traits having an adaptive function in response to their native environment (leaves of a similar age were collected from *E. camaldulensis* and *E. globulus* trees grown under similar environmental conditions). Cuticular differences between species belonging the same genus have been previously reported with especial focus on EW (e.g., Hallam and Chambers, 1970; Riedel et al., 2007).

## ASSESSING THE LINK BETWEEN CUTICULAR CHEMISTRY AND ULTRA-STRUCTURE

### SOXHLET EXTRACTED CUTICLES

The lamellate CP regions observed in intact leaf cuticles were replaced by amorphous, electron-lucent bands after Soxhlet extraction. These bands had a thickness in the same range or slightly below that of the corresponding intact CP. By contrast, no remarkable changes were noticed between the CL of intact and SE cuticles. X-ray diffraction and TEM studies performed on *Hedera helix* leaf cuticles both intact and after solvent extraction revealed similar results, suggesting an intermolecular disorganization of the cuticles, particularly in the CP (Viougeas et al., 1995). The appearance of the CL was also preserved (Viougeas et al., 1995), alike in *Agave americana* extracted cuticles (Wattendorff and Holloway, 1982). The CP thickness variation may be due to several phenomena which may also affect the CL region, such as the loss of cuticular material and structure, or the potential shrinkage of the tissues as affected by Soxhlet extraction or TEM sample preparation procedures. The influence of cuticle position within one or between different leaves should also be considered (Riederer and Schönherr, 1988).

No clear relationship between soluble lipids composition and cuticle internal morphology could be established. As an exception, *E. camaldulensis* epicuticular plates may correspond to hentetracontan-1-ol (Jeffree, 2006). The chemical nature of the CP lamellae has been attempted to be identified in a few investigations (see Jeffree, 2006), but remains unclear so far. Our results indicate that the electron-lucent lamellae of *E. camaldulensis* and *E. globulus* CP are partially composed of soluble lipids as suggested by Domínguez and Heredia (1999) for the *Clivia miniata* leaf cuticle, but the presence of other free or chemically bound compounds cannot be discarded. The granular structures covering the inner, intact cuticle surface which were removed during Soxhlet extraction may be associated with soluble intracuticular lipids. The remaining granules may correspond to insoluble lipids and/or cutin (i.e., cutinsomes; Heredia-Guerrero et al., 2008).

### DE-ESTERIFIED RESIDUES

The de-esterification step led to substantial structural changes as compared to the Soxhlet extraction process. While FTIR spectra confirmed the major polysaccharide nature of the SED residues (Heredia-Guerrero et al., 2014), TEM micrographs enabled us to observe the cellulose architecture of the external cell wall, with a non-ordered, outer region and a larger multi-layered, helicoidal inner region (also visible in cuticular pegs). The relative position within the cuticles and measurement of TEM micrographs of intact, SE cuticles and such SED residues indicate that these regions may be ascribed to the CP and the CL, respectively.

One or the two cellulose fibril orientation patterns found in the SED residues have been previously reported for the epidermal cell wall of a few species and organs (e.g., Roland et al., 1982; Kutschera, 2008), and also for the *H. helix* leaf cuticle (Canet et al., 1996). Thereby, the structure of the cellulose network seems to leave an imprint on the morphology of cuticular cross-sections and may contribute to the different ultra-structure of the CP (possibly with randomly oriented cellulose) and CL (with ordered cellulose). However, it is also possible that the removal of cuticular material may have disrupted the orientation and arrangement of the cellulose network. Other factors should be also taken into account, such as those affecting SE cuticles.

Together with cellulose, it is likely that other cuticular polysaccharides, such as pectins or hemicelluloses, extend throughout the cuticle, as derived from cellulose and pectin distribution in intact leaf cuticles of three model species (Guzmán et al., 2014b). Under certain conditions, these polysaccharides might form a gel-like phase with embedded cellulose fibrils, as described for plant cell walls (Cosgrove, 2005; Burton et al., 2010). By analogy with the cell wall, the cellulose fibrils may constitute the reinforcing rods of the cuticle, while the non-cellulosic phase may provide flexibility, mechanical support (Burton et al., 2010), and facilitate the transport of hydrophilic, polar compounds and water through the cuticle (Guzmán et al., 2014a,b).

A significant amount of material was removed largely from the inner cuticle region, which experienced a thickness reduction of approximately 60–80%. Working with *A. americana* leaf cuticles, Wattendorff and Holloway (1982) reported a thickness reduction of 40% or more from the inner side after cutin depolymerization. Apart from the lower content and/or degree of polymerization of cutin located in the more exposed internal CL, which may yield this region more susceptible to degradation (Wattendorff and Holloway, 1982), the role of further chemical compounds should also be considered as discussed above.

### IMPLICATIONS OF THE RESULTS OBTAINED

The chemical and structural analyses performed with *E. globulus* and *E. camaldulensis* leaves, indicate that the cuticle of such species may be interpreted as a lipidized cell wall region. This result is in agreement with the viewpoint of Yeats and Rose (2013), who suggested that the cuticle can be considered a specialized lipidic modification of the cell wall. Our results show that a polysaccharide network may provide the basis for cuticle structure, a role which has been frequently assigned almost exclusively to cutin (Kolattukudy, 1970). Cuticular lipid constituents may impregnate and be intruded into such cell wall matrix, with waxes being also deposited on to the outer cuticle surface, early at epidermal cell ontogeny (Jeffree, 2006; Guzmán et al., 2014b). The occurrence of a polysaccharide network throughout the entire cuticle, with the exception of the EW layer, may have significant bio-mechanical implications and also contribute to the multi-directional transport of water and solutes as suggested by Guzmán et al. (2014a,b).

It is likely that the actual proportion of polysaccharides is seriously underestimated by current methods of cuticular handling and analysis. The importance of polysaccharides as integral, essential cuticle materials should be recognized for the development of methodologies which allow a proper interpretation of the plant cuticle in biological, chemical, and physical terms. In light of the present study, the prevailing model which conceives the cuticle as a lipidic and hydrophobic extra-cellular layer should be reconsidered, highlighting its cell wall nature and heterogeneous character in terms of polarity/apolarity and hydrophobicity/hydrophilicity. However, further studies with different plant organs, organ parts, and species will be required for proposing a broader plant cuticle model, and also for establishing a link between cuticle chemistry, structure, and function under an ecophysiological perspective.

While no specific cuticle structural patterns could be identified in association with chemical composition, the background cell wall architecture seems to have an effect on cuticle morphology. We hence conclude that the existing methodologies do not fully enable to establish a direct link between cuticle composition and structure. Therefore, the implementation of new experimental approaches also analyzing the performance of pure chemical compounds (e.g., cutin monomers) may help us elucidate the link between cuticle structure and chemistry also in relation with different plant species, organs, and habitats.

## Conflict of Interest Statement

The authors declare that the research was conducted in the absence of any commercial or financial relationships that could be construed as a potential conflict of interest.
